# Colonization of root cells and plant growth promotion by *Piriformospora indica* occurs independently of plant common symbiosis genes

**DOI:** 10.3389/fpls.2015.00667

**Published:** 2015-09-17

**Authors:** Aline Banhara, Yi Ding, Regina Kühner, Alga Zuccaro, Martin Parniske

**Affiliations:** ^1^Faculty of Biology, Institute of Genetics, University of MunichMartinsried, Germany; ^2^Department of Organismic Interactions, Max Planck Institute for Terrestrial MicrobiologyMarburg, Germany; ^3^Cluster of Excellence on Plant Sciences, Botanical Institute, University of CologneCologne, Germany

**Keywords:** *Piriformospora indica*, *Lotus japonicus*, *Arabidopsis thaliana*, biotrophy, common symbiosis genes, intracellular colonization, growth promotion

## Abstract

Arbuscular mycorrhiza (AM) fungi (Glomeromycota) form symbiosis with and deliver nutrients via the roots of most angiosperms. AM fungal hyphae are taken up by living root epidermal cells, a program which relies on a set of plant common symbiosis genes (CSGs). Plant root epidermal cells are also infected by the plant growth-promoting fungus *Piriformospora indica* (Basidiomycota), raising the question whether this interaction relies on the AM-related CSGs. Here we show that intracellular colonization of root cells and intracellular sporulation by *P. indica* occurred in CSG mutants of the legume *Lotus japonicus* and in *Arabidopsis thaliana*, which belongs to the Brassicaceae, a family that has lost the ability to form AM as well as a core set of CSGs. *A. thaliana* mutants of homologs of CSGs (HCSGs) interacted with *P. indica* similar to the wild-type. Moreover, increased biomass of *A. thaliana* evoked by *P. indica* was unaltered in HCSG mutants. We conclude that colonization and growth promotion by *P. indica* are independent of the CSGs and that AM fungi and *P. indica* exploit different host pathways for infection.

## Introduction

Plants form mutualistic interactions with fungi from different taxonomic groups. Arbuscular mycorrhiza (AM) is a widespread symbiosis between plants and fungi of the phylum Glomeromycota (Parniske, 2008; Smith and Read, 2008) and is considered a key factor that allowed plants to colonize land more than 400 million years ago (Schüßler and Walker, 2011). The ancestral nature of this interaction raises the question to what extent other and potentially younger interactions between plant roots and fungi evolved by co-opting the genetic framework for AM formation. Candidates for such interactions include endomycorrhizal interactions formed with fungi of the order Sebacinales of the phylum Basidiomycota (Selosse et al., 2009).

An experimental model for this group of fungi is *Piriformospora indica*, which infects various taxonomically unrelated hosts and can increase plant growth and biomass (Peškan-Berghöfer et al., 2004; Waller et al., 2005; Shahollari et al., 2007; Sherameti et al., 2008; Camehl et al., 2010, 2011; Hilbert et al., 2012; Nongbri et al., 2012; Lahrmann et al., 2013; Venus and Oelmüller, 2013).

Phythormones like ethylene, jasmonic acid and gibberellins seem to positively influence root colonization by *P. indica*, while salicylic acid has an inhibitory effect (Jacobs et al., 2011; Khatabi et al., 2012). Genes involved in the synthesis of indole-3-acetaldoxime-derived compounds restrict the growth of *P. indica* within *Arabidopsis thaliana* roots (Nongbri et al., 2012). Indole-3-acetaldoxime is also an intermediate in the biosynthesis of the phytohormone indole-3-acetic acid (IAA) (Mikkelsen et al., 2009; Burow et al., 2010), which has been implicated in the *P. indica*-induced host growth promotion, together with cytokinin (Vadassery et al., 2008).

In the initial stages of plant root colonization, *P. indica* invades living plant cells (Jacobs et al., 2011; Zuccaro et al., 2011; Lahrmann and Zuccaro, 2012; Lahrmann et al., 2013). This initial stage is followed by the death of colonized cells even though the plant host displays no macroscopic signs of disease (Deshmukh et al., 2006; Jacobs et al., 2011; Zuccaro et al., 2011; Lahrmann and Zuccaro, 2012; Qiang et al., 2012). In *A. thaliana* roots, *P. indica* continuously infects cells *de novo* and colonized living cells are found beside dying and dead colonized cells 3 days after infection (Qiang et al., 2012).

During the early stage of the interaction, a plant-derived membrane has been observed which surrounds and separates *P. indica* hyphae from the plant cytoplasm (Jacobs et al., 2011; Lahrmann and Zuccaro, 2012; Lahrmann et al., 2013). The structural arrangement of this interaction is similar to that of transcellular and arbuscular hyphae of AM fungi, which are equally surrounded by a peri-fungal membrane (Bonfante and Genre, 2010).

We explored whether these structural similarities are reflected by a shared genetic basis between both symbioses. The successful invasion of the outer cell layers by AM fungi requires an ancestral plant genetic program that is conserved among angiosperms and encompasses the common symbiosis genes (CSGs). In legumes, the CSGs are required for both AM and the root nodule symbiosis with nitrogen-fixing bacteria (Kistner et al., 2005; Gutjahr, 2014; Svistoonoff et al., 2014), and encode the symbiosis receptor-like kinase SYMRK (Antolín-Llovera et al., 2014; Ried et al., 2014), the nucleoporins of the NUP107–160/NUP84 subcomplex (Alber et al., 2007) NUP85, NUP133, and the SEC13 HOMOLOG1 (SEH1) NENA (Kanamori et al., 2006; Saito et al., 2007; Groth et al., 2010), CASTOR and POLLUX, cation channels localized at the nuclear envelope (Charpentier et al., 2008; Venkateshwaran et al., 2012), as well as the nucleoplasmatic complex formed by a calcium and calmodulin-dependent protein kinase (CCaMK) and CYCLOPS responsible for calcium signal decoding (Singh and Parniske, 2012; Singh et al., 2014). The signal transduction pathway involving the products of the CSGs leads from the perception of microbial signals at the plasma membrane to the transcriptional activation of genes in the nucleus (Gutjahr and Parniske, 2013). In the legume *Lotus japonicus* CSG mutants are all impaired in the intracellular accommodation of both rhizobia and AM fungi (Kistner et al., 2005).

*A. thaliana* is a member of the Brassicaceae, a family which lost the ability to establish AM symbiosis (Delaux et al., 2014). This loss is correlated with the absence of a specific set of CSGs, including *CCaMK* and *CYCLOPS* (Delaux et al., 2014). Despite this loss, *A. thaliana* retained homologs of common symbiosis genes (HCSGs). Based on phylogenetic and/or synteny analyses, orthologs or candidate orthologs of legume CSGs in *A. thaliana* have been identified for *POLLUX* (Ané et al., 2004; Delaux et al., 2013) and genes coding for members of the NUP107–160 subcomplex, including *NUP133* and *SEC13* (Wiermer et al., 2012; Binder and Parniske, 2013). Importantly, an *AtPOLLUX* version was able to restore nodulation in the non-nodulating mutant *dmi1–4* of *M. truncatula* (Venkateshwaran et al., 2012), indicating relative conservation of its symbiotic activity in *A. thaliana*. *SYMRK* encodes a malectin-like domain leucine-rich repeat receptor-like kinase, a gene family that has expanded to 50 members in *A. thaliana* (Hok et al., 2011). However, based on synteny analysis (Kevei et al., 2005) and the lack of SYMRK-specific amino-acid sequence patterns in the kinase domain (Markmann et al., 2008), a SYMRK ortholog appears to be absent from the *A. thaliana* genome. ShRK1 (SYMRK-homologous Receptor-like Kinase 1) and ShRK2 are the closest SYMRK homologs in *A. thaliana*. The domain organization of ShRK1 and 2 is identical to that of *L. japonicus* SYMRK (Markmann et al., 2008).

To explore the role of *L. japonicus* CSGs and *A. thaliana* HCSGs in the interaction with *P. indica*, we investigated whether the fungus was able to penetrate root cells, complete its life cycle and promote host plant growth in the respective mutant backgrounds.

## Materials and methods

### Fungal strains and growth conditions

*P. indica* (Verma et al., 1998, DSM11827, Leibniz Institute DSMZ—German Collection of Microorganisms and Cell Cultures, Braunschweig, Germany) and *Piriformospora williamsii* (Basiewicz et al., 2012) were grown at 28°C in the dark on plates with solid (1.5% agar) complete medium (CM) (Pham et al., 2004). For studies with plants grown in soil, fungal mycelium was propagated in liquid CM medium, in the dark, at RT and 120 rpm shaking. Mycelium was washed three times with sterile distilled water directly prior to mixing with soil substrate.

For experiments on plates, fungal chlamydospores were obtained from 4 to 6-week-old cultures, as follows: Tween water (0.002% Tween 20) was added to plates containing mycelium, which was rubbed off from the agar surface, and the resulting spore suspension was collected and centrifuged three times at 3000 *g* for washing. Chlamydospore concentration was estimated using a Fuchs–Rosenthal counting chamber and adjusted to the desired 5 × 10^5^ ml^−1^ with Tween water.

### Plant genotypes and growth conditions

For co-cultivation with *P. indica, L. japonicus* seeds (Supplementary Table 1) were surface sterilized in a 2% sodium hypochlorite solution for 7 min, washed three times with sterile water for 5 min each, and imbibed in water overnight. Seeds were then put on plates with solid modified Hoagland's medium [HO, 5 mM KNO_3_; 5 mM Ca(NO_3_)_2_; 2 mM MgSO_4_; 4 mM KH_2_PO_4_; 0.03 g l^−1^ Sprint 138 iron chelate; 0.1% micronutrients solution containing 2.86 g l^−1^ H_3_BO_3_; 1.81 g l^−1^ MnCl_2_.4H_2_O; 0.08 g l^−1^ CuSO_4_.5H_2_O; 0.02 g l^−1^ 85% MoO_3_.H_2_O; based on Hoagland and Arnon (1950)] and kept for 4 days at 24°C in the dark for germination.

*A. thaliana* seeds were obtained from “The Nottingham *A. thaliana* Stock Centre”—NASC (Scholl et al., 2000) (Supplementary Table 2). For co-cultivation with *P. indica* or *P. williamsii* and tests for plant growth promotion, *A. thaliana* seeds were sterilized by incubation for 5 min in 70% ethanol, 0.05% Tween 20 followed by 2 min in 100% ethanol, left to dry and stratified for 48 h at 4°C in the dark. Plants were then grown under long day conditions (16 h:8 h, light:dark, at 23°C, 85 μmol.m^−2^ s^−1^), on half-strength (½) Murashige and Skoog medium (MS, Murashige and Skoog, 1962) with or without sucrose (0.05%) or on modified HO solidified with gelrite 4 g l^−1^, and four times more phosphate than in the original recipe.

Seven to ten-day-old *A. thaliana* or four-day-old *L. japonicus* plants were mock-inoculated with 1 ml Tween water (control) or either *P. indica* or *P. williamsii* chlamydospore suspensions. Non-germinated seeds and retarded seedlings were removed from the plates before inoculation. Co-cultures or mock-inoculated plants were grown at 24°C under long day conditions (16 h:8 h, light:dark). Biomass of seedlings was determined using a digital microbalance 7 days post inoculation (dpi).

For analysis of growth promotion under different nutrient regimes, plants were grown on the following media: modified HO with no KNO_3_ or Ca(NO_3_)_2_, supplemented with 0.05 mM KNO_3_, 0.5 mM KNO_3_, or 5 mM KNO_3_ (standard concentration), modified HO depleted of phosphate or with 4 mM KH_2_PO_4_(standard concentration); and modified HO depleted of ammonium or with 10 or 20 mM NH_4_Cl. Where necessary, potassium and calcium concentrations were compensated with KCl and CaCl_2_, respectively.

For growth promotion experiments in soil, plants were grown on either low-nutrient soil (50–100 mg l^−1^ N, 50–100 mg l^−1^ P, 100–150 mg l^−1^ K) or high-nutrient soil (500 mg l^−1^ N, 500 mg l^−1^ P, 500 mg l^−1^ K) mixed with 1 g fresh or autoclaved mycelium per 100 g sterile substrate for inoculation and mock-inoculation, respectively. Plant height was determined 7, 11, 16, and 21 dpi.

### Sequence alignments

Complete protein sequences were obtained from The Arabidopsis Information Resource (TAIR—www.arabidopsis.org) for *A. thaliana*, and from the GenBank for *L. japonicus*. Alignments (Supplementary Figures 7–9) were performed with MAFFT 6.822 (Katoh et al., 2002) or ClustalW2 (Larkin et al., 2007; Goujon et al., 2010) with the default settings. Searches for conserved domains in the protein sequences were performed using ScanProsite (de Castro et al., 2006) and/or InterProScan (Jones et al., 2014), and based on data available from Kanamori et al. (2006), Markmann et al. (2008), and Groth et al. (2010).

### Determination of fungal colonization by microscopy

To observe *P. indica* intracellular sporulation in *A. thaliana* and *L. japonicus* roots, colonized roots at 14 dpi were incubated at 96°C for 1 (*A. thaliana*) or 10 min (*L. japonicus*) in 10% (w/v) KOH and double-stained in the dark at room temperature for 20 min with 10 μg ml^−1^ WGA-AF488 (Wheat Germ Agglutinin-Alexa Fluor 488) (Molecular Probes, Karlsruhe, Germany) to visualize fungal structures, and 10 μg ml^−1^ propidium iodide (PI) to visualize plant cell walls. Samples were analyzed with a Leica DMI6000B microscope using differential interference contrast or epifluorescence (GFP filter set for WGA-AF488: excitation 450–490 nm, emission 500–550 nm; TX2 filter settings for PI: 540–580 nm excitation, 608–683 nm emission).

Root colonization and plant cell viability were analyzed by confocal laser scanning microscopy (CLSM). Fungal cell walls within colonized roots at 4 (*A. thaliana*), and 3 dpi (*L. japonicus*) were affinity-labeled for 10 min with 10 μg ml^−1^ WGA-AF488 (Molecular Probes, Karlsruhe, Germany). Membranes were stained with 3 μM FM4-64 (Molecular Probes, Karlsruhe, Germany) for 4 min (at 260 mm Hg). Root samples were imaged with TCS-SP5 or TCS-SP8 confocal microscopes (Leica, Bensheim, Germany) with excitation at 488 nm for WGA-AF488 and detection at 500–540 nm. FM4-64 was excited at 633 nm and detected at 650–690 nm. Propidium iodide was excited at 540 nm and detected at 600–630 nm.

### Quantification of fungal colonization by qPCR

Roots of *A. thaliana* and *L. japonicus* colonized with *P. indica* (14 dpi) were thoroughly washed to remove fungal hyphae from the root surface. Two hundred micrograms of root material were then used for DNA extraction according to the protocol of Doyle and Doyle (1987). Real-time qPCR analyses were performed from 10 ng DNA mixed with the appropriate primers: *P. indica Transcription Elongation Factor* (Butehorn et al., 2000); *A. thaliana Ubiquitin* (Khatabi et al., 2012); or *L. japonicus Ubiquitin* (Takeda et al., 2009) in 10 μl SYBRgreen Supermix (BIORAD) using the following amplification protocol: 2′–95°C; 40 × (30″–95°C; 30″–59°C; 30″–72°C); melting curve 95°C–60°C–95°C. Fungal colonisation was quantified by the 2^−△^^*Ct*^ method (Livak and Schmittgen, 2001) by subtracting the raw threshold cycle (Ct) values of *P. indica* TEF from those of plant UBI to obtain Δ*Ct*.

### β-glucuronidase (GUS) staining assays

GUS staining assays were performed with the *L. japonicus* symbiosis-reporter line T90 (Webb et al., 2000). Colonized roots at 3, 7, and 14 dpi with *P. indica* chlamydospores or Tween water were vacuum infiltrated with X-Gluc staining solution (100 mM sodium phosphate buffer pH 7.0; 10 mM EDTA; 0.1% Triton-X 100; 0.5 mg ml^−1^ X-Gluc; 1 mM K_3_[Fe(CN)_6_]; 1 mM K_4_[Fe(CN)_6_].3H_2_O) three times for 10 min, followed by incubation at 37°C for 18 h in the dark. Root systems were inspected for GUS staining with a Leica MZFLIII stereomicroscope.

The *Mesorhizobium loti* MAFF303099 strain constitutively expressing *Ds*RED (Maekawa et al., 2009) was used to inoculate roots of the *L. japonicus* T90 line as a suspension in Fahraeus medium (Fahraeus, 1957) adjusted to an OD_600_ of 0.05.

### Statistical analyses

Statistical analyses were performed with R version 3.0.2 (2013-09-25) “Frisbee Sailing” (R Core Team, 2013) using the package “agricolae” (De Mendiburu, 2010). Pairwise or multiple comparisons of the different subsets of data were performed with the Kruskal–Wallis test followed by a Bonferroni–Holm correction using the mock-inoculated samples as control group. Two-sided, unpaired *t*-tests with equal variance were used to analyze relative amount of fungal DNA within plant roots.

## Results and discussion

### Intracellular infection and sporulation by *P. indica* occurs independently of *L. japonicus* and *A. thaliana* common symbiosis genes

**Figure 1:**
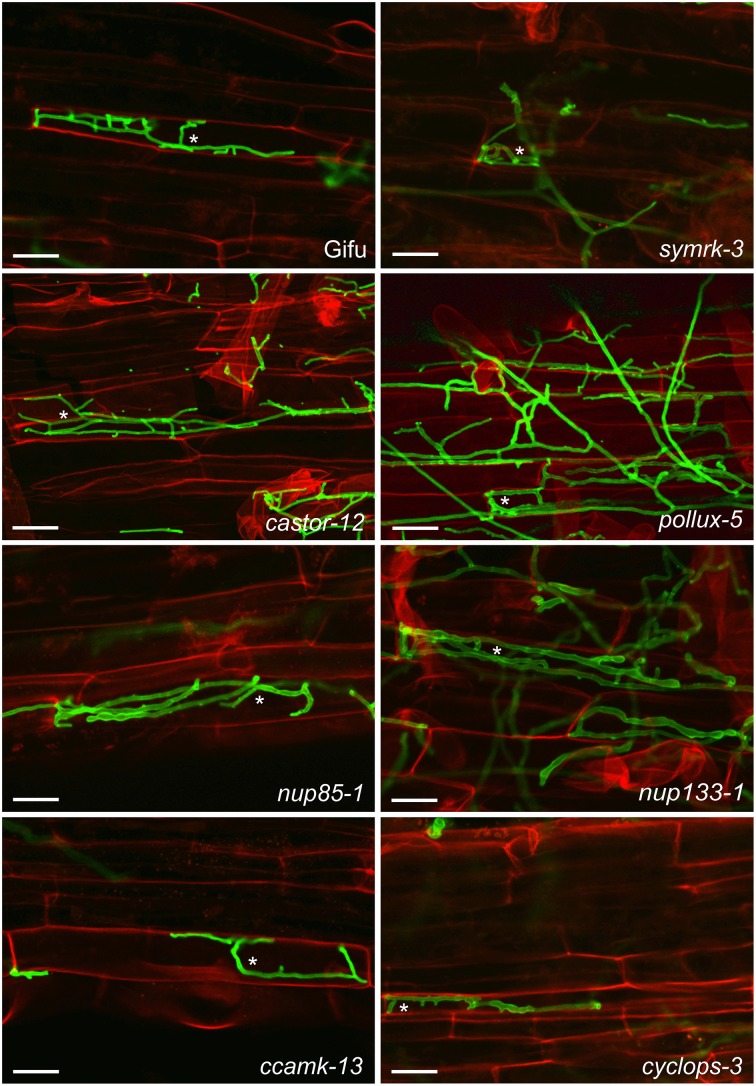
**Colonization of ***L. japonicus*** root cells by ***P. indica*****. Wild-type (Gifu) and the indicated common symbiosis mutants were analyzed 7 dpi with *P. indica* chlamydospores. Intracellular hyphae were present in all genotypes (examples are indicated by asterisks). Roots were cleared and double stained with propidium iodide (red), for cell wall visualization, and WGA-AF488 (green), for fungal structures. Scale bar: 25 μm.

We investigated whether the classical CSGs are involved in the interaction between roots of the legume *L. japonicus* and *P. indica*. In root epidermal cells of *L. japonicus* wild-type (ecotype “Gifu”), and in CSG mutants intracellular hyphae were detected at 7 dpi (Figure 1) and sporulation at 14 dpi (Supplementary Figure 1), evidencing that *P. indica* entered host cells and successfully completed its life cycle in all genotypes tested. These observations indicate that the CSGs are not required for the successful infection of host cells by *P. indica*.

The *L. japonicus* symbiosis-reporter line T90 carries a promoter:*GUS* fusion and is activated in response to *Mesorhizobium loti* or nodulation factors, and AM fungi (Webb et al., 2000; Radutoiu et al., 2003; Kistner et al., 2005). T90 reporter activation requires *L. japonicus* CSGs (Kistner et al., 2005; Gossmann et al., 2012) and dominant active alleles of *SYMRK* and *CCaMK* are sufficient for T90 induction (Ried et al., 2014 and unpublished data). However, we did not detect GUS activity (blue staining) in *P. indica* or mock-inoculated roots, while blue staining was observed in *M. loti Ds*Red inoculated roots (Supplementary Figure 2). These data indicate that the promoter:GUS fusion of the T90 line and the signal transduction pathway upstream are not activated during the interaction of *L. japonicus* with *P. indica*.

**Figure 2:**
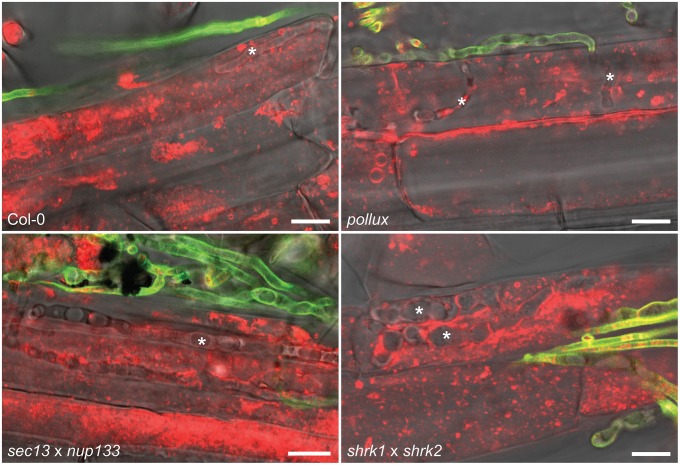
**Colonization of ***A. thaliana*** root cells by ***P***. ***indica*****. Hyphae (indicated by asterisks) were detected 4 dpi with *P. indica* chlamydospores within root cells of wild-type (Col-0) and the indicated HCSG mutants. Extracellular hyphae were stained with WGA-AF488 (green) but intracellular hyphae were not or weakly fluorescent, probably due to limited access of WGA-AF488 to the fungal cell wall within root cells. FM4-64-stained plant material (red) within invaded host cells is indicative of stage 1 or 2 of the infection process. Scale bar 10 μm.

Moreover, we observed that *A. thaliana* wild-type (Col-0), which lacks some of the key CSGs (Delaux et al., 2014), supported intracellular root colonization and sporulation by *P. indica* (Figure 2 and Supplementary Figure 3). In addition, intracellular infection and sporulation also occurred in the roots of the *A. thaliana* HCSG mutants *pollux, nup133, sec13*, and the double mutants *sec13* × *nup133* and *shrk1* × *shrk2* (Figure 2 and Supplementary Figure 3), indicating that the fungus could successfully infect these mutants and complete its life cycle. These observations strongly support the conclusion that neither the CSGs which *A. thaliana* lost during its evolution nor the experimentally mutated HCSGs are required for the interaction with *P. indica*.

### Growth of *P. indica* within *L. japonicus* and *A. thaliana* root cells

Fungal structures were detectable within the boundaries of root epidermal cells in both *L. japonicus* (Figure 1 and Supplementary Figure 1) and *A. thaliana* (Supplementary Figure 3). We investigated whether these hyphae would penetrate into living plant cells. To determine the vitality status of the root cells, we used the lipophilic stain FM4-64 in combination with time-lapse imaging. After FM4-64 staining, *P. indica* hyphae were observed within cells containing mobile structures including possible vesicles in *A. thaliana* (Figure 2). In this host, we could detect three states of colonized root cells that differed in the mobility or presence of intracellular content. In the first state, vesicle-like structures moved at a speed similar to that observed within neighboring non-infected cells (Supplementary Movie 1) or cells of non-colonized roots (Supplementary Movie 3). In exceptional cases, some of these cells contained small, intact vacuoles. In the second state, the FM4-64-stained material showed very little or no movement (Supplementary Movie 2), and occasionally collapsed vacuoles were observed. We also observed invaded cells with no cytoplasmic content, probably representing a third state of cellular infection, during which the fungus grows within likely dead cells (Supplementary Figure 4, Supplementary Movie 2). This observation supports a three-stage model of cellular infection, in which *P. indica* first colonizes individual roots cells that show vesicular movement. In stage 2, vesicular movement has undergone at least partial arrest. In stage 3 the cellular content has disappeared, possibly through autophagocytosis or consumption by the fungus (Supplementary Figure 4). Immobilized, irregular fluorescent structures are also present within probably dying cells of non-colonized roots (Supplementary Movie 3, white arrowhead). Such dying cells have been attributed to a developmental program implicated in developmental events such as the removal of root cap cells (Fendrych et al., 2014).

Movement of FM4-64-labeled material in *P. indica*-infected cells could also be documented in the *A. thaliana* HSCG mutant *pollux* (Supplementary Movie 4), and the double mutants *sec13* × *nup133* and *shrk1* × *shrk2*, indicating that similar stages of cell activity occur independently of HCSGs. Because of the technically demanding process of obtaining such movies, a quantitative comparison of colonization stages between the wild-type and the HCSG mutants was not performed.

### Increased relative fungal biomass within roots of *L. japonicus* common symbiosis mutants

**Figure 3:**
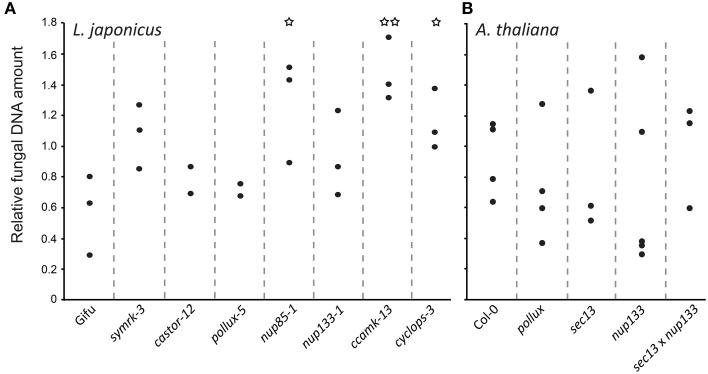
**Quantification of ***P. indica*** in ***L. japonicus*** (A) and ***A. thaliana*** (B) wild-type and mutant roots**. Real-time qPCR was used to quantify DNA from surface-washed *P. indica*-colonized roots at 14 dpi grown on modified HO medium using primers for the fungal gene *Transcription Elongation Factor* (*TEF*) and for *A. thaliana* and *L. japonicus Ubiquitin* (*UBI*) genes. Differences between the wild-type and mutants were investigated with a two-sided, unpaired *t*-test. ^*^P < 0.05, ^**^P < 0.01.

In order to quantify the relative fungal biomass within host roots, we determined the ratio between fungal DNA and plant DNA. Interestingly, in *L. japonicus*, we detected a tendency for an increased relative amount of fungal DNA in most of the tested common symbiosis mutants, with a significant difference to the wild-type in *nup85-1, ccamk-13*, and *cyclops-3* (Figure 3). This higher ratio of fungal to plant DNA may be the result of increased fungal proliferation, reduced root growth, and/or plant cell death in the mutants. Curiously, this effect was only observed on *L. japonicus* but not on *A. thaliana* mutants (Figure 3). Our results reveal that CSG-mediated pathways affect the relative *P. indica* colonization level in *L. japonicus*, and that this regulation is not operational in *A. thaliana*. Interestingly, CSGs potentially have a cell protecting effect (Esseling et al., 2004; Genre et al., 2009; Evangelisti et al., 2014). Apart from its signaling role in symbiosis (Antolín-Llovera et al., 2014; Ried et al., 2014), *SYMRK* has been implicated in desensitization of root hair cells against mechanic stress (Esseling et al., 2004), and *CCaMK* increased tolerance against the cell killing effect of *Colletotrichum* (Genre et al., 2009). In a *ccamk* mutant of *M. truncatula* infected by the hemi-biotrophic fungal pathogen *Colletotrichum trifolii*, the switch from biotrophy to necrotrophy occurred earlier (Genre et al., 2009).

In barley, a dense colonization by *P. indica* is associated with root cell death (Deshmukh et al., 2006; Camehl et al., 2010; Nongbri et al., 2012). There is evidence that excessive *P. indica* proliferation is associated with cell death and/or with detrimental effects on the growth of the plant host (Deshmukh et al., 2006; Camehl et al., 2010; Nongbri et al., 2012). It is therefore possible that *L. japonicus ccamk* mutants suffer from an earlier onset of the necrotrophic phase of *P. indica* colonization, and that CSGs contribute to the maintenance of plant cellular integrity after fungal invasion. On the other hand, the CSGs are critical for lipochitooligo-saccharide (LCO)-induced lateral root emergence (Oláh et al., 2005; Maillet et al., 2011). While it is unclear whether *P. indica* stimulates lateral root emergence and, if so, whether it does it via the common symbiosis pathway, the altered fungal to plant biomass ratio of the CSG mutants could be due to a reduction in host root proliferation.

### Plant growth promotion by *P. indica* is influenced by nutrient availability

**Figure 4:**
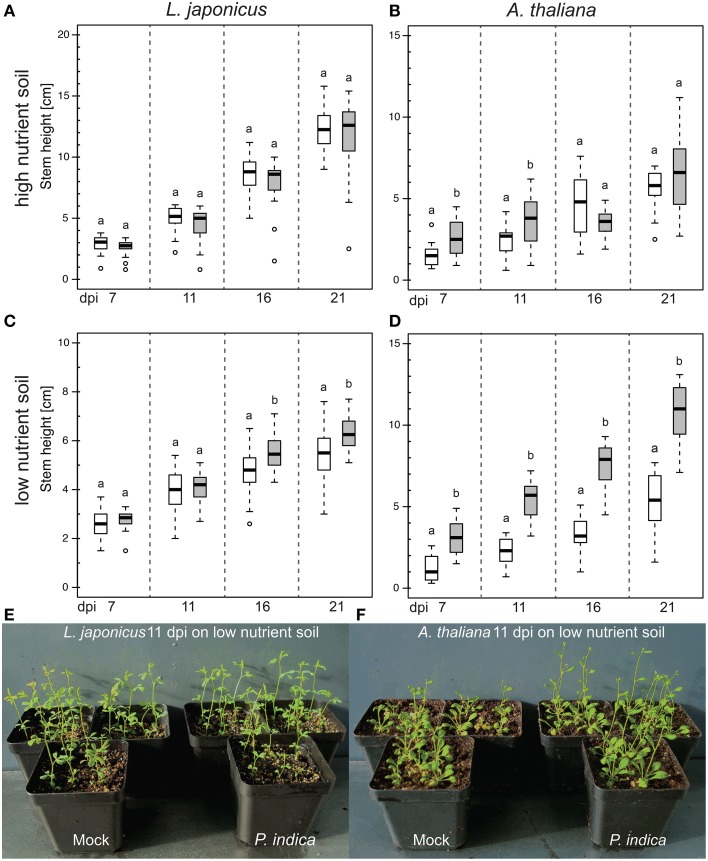
**Effect of ***P. indica*** on the stem height of ***L. japonicus*** and ***A. thaliana*** grown in high or low nutrient soil**. Box-plots represent the stem height of ca. 20 plants per treatment at the indicated dpi. **(A,B)** In high-nutrient soil (500 mg l^−1^ N, 500 mg l^−1^ P, 500 mg l^−1^ K), inoculation with *P. indica* did not change plant stem height at 21 dpi (*p* > 0.05). **(C,D)** On low-nutrient soil (50–100 mg l^−1^ N, 50–100 mg l^−1^ P, 100–150 mg l^−1^ K), *P. indica* inoculation led to an increase in the mean stem height at 21 dpi (*p* < 0.05). Statistical analyses were performed with a Kruskal–Wallis test followed by a Bonferroni–Holm correction using the mock-inoculated plants as control group. For each mock/*P. indica*-inoculated pair, box-plots sharing the same letter do not significantly differ (at the 5% significance level). White boxes: mock; gray boxes: *P. indica*-inoculated; open circles: outliers. (**E,F**) Exemplary pictures of *P. indica*- and mock-inoculated plants grown in low nutrient soil. Experiments were performed three times with similar results.

In order to obtain an experimental system for the genetic dissection of the *P. indica*-mediated growth promotion (Peškan-Berghöfer et al., 2004; Shahollari et al., 2007; Sherameti et al., 2008; Camehl et al., 2010, 2011; Nongbri et al., 2012; Lahrmann et al., 2013; Venus and Oelmüller, 2013), we explored the influence of the substrate and nutrient availability. We evaluated the effect of *P. indica* on *L. japonicus* and *A. thaliana* grown in soil with two different nutrient concentrations. In both plant species, co-cultivation with *P. indica* in soil with high nutrient concentrations had little or no effect on the mean stem height. However, in soil with lower nutrient contents, *P. indica* inoculation roughly doubled the mean stem height of *A. thaliana* plants at 21 dpi, whereas there was only a small but significant effect on *L. japonicus* (Figure 4).

When *A. thaliana* plants were grown on agar plates with ½MS medium and no sugar, and inoculated with chlamydospores of *P. indica*, a growth promoting effect was observed. In contrast, when 0.05% sucrose was added to the medium, the plants were generally bigger and no increase was observed in the mean fresh weight of *P. indica*-inoculated plants compared to mock-treated plants (Supplementary Figure 5).

Since *A. thaliana* plants grown in the presence of *P. indica* exhibit increased uptake of nitrogen and phosphate (Shahollari et al., 2007; Kumar et al., 2011; Das et al., 2014), we investigated whether the concentration of nitrate [supplied as Ca(NO_3_)_2_ and/or KNO_3_], phosphate (KH_2_PO_4_), or ammonium (NH_4_Cl) on modified HO medium influenced the growth promotion of *A. thaliana* plants by *P. indica*. Co-cultivation with the fungus had a positive effect on the mean fresh weight of the plants under all nutrient conditions tested, except on a medium that limited plant growth due to the lack of a nitrogen source (Supplementary Figure 6). For the subsequent experiments, including those already reported in Lahrmann et al. (2013), we used modified HO medium, which consistently supported the growth-promoting effect by *P. indica*.

### *A. thaliana* homologs of common symbiosis genes are not required for *P. indica*-induced growth promotion

**Figure 5:**
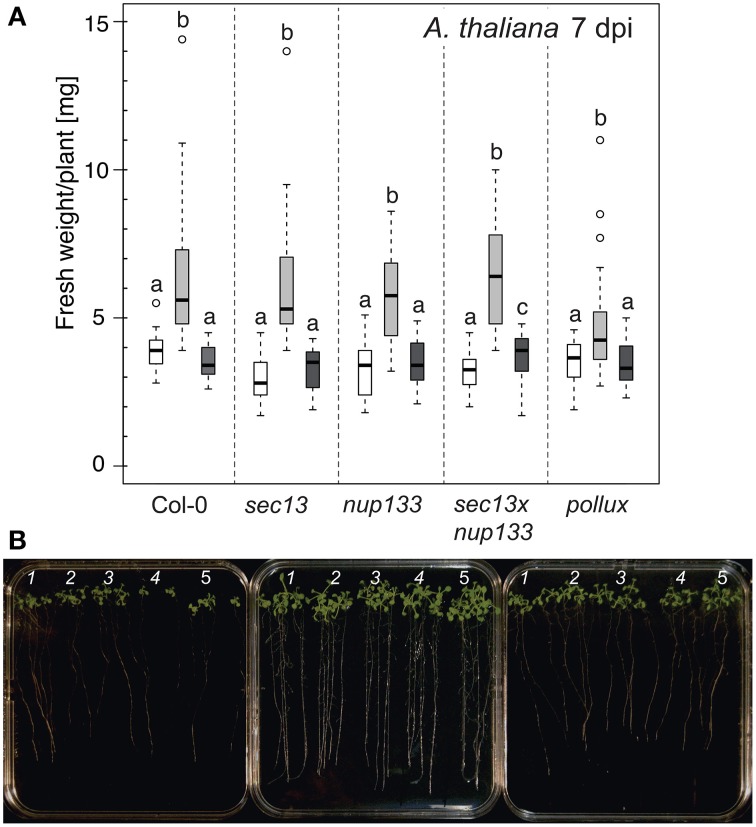
**Effect of ***P. indica*** or ***P. williamsii*** on the biomass of ***A. thaliana*****. **(A)** Box-plots show the fresh weight of the indicated *A. thaliana* genotypes (ca. 15 plants/genotype) grown in the presence or absence of *P. indica* or *P. williamsii*. All plant genotypes accumulated more biomass upon co-cultivation with *P. indica* but not with *P. williamsii*. White box: mock; light gray box: *P. indica*; dark gray box: *P. williamsii*. Open circles: outliers. Statistical analyses were performed using a Kruskal–Wallis test followed by a Bonferroni–Holm correction using the mock-inoculated samples as control group. Groups that do not share the same letter are significantly different (at the 5% significance level). Comparisons between the three treatments were made for each genotype separately. Plant biomass was determined 7 dpi. **(B)** Representative plates showing sets of 14-day-old plants grown on modified HO medium are shown 7 days after mock-treatment with Tween water (left) or inoculation with either *P. indica* (center) or *P. williamsii* (right) chlamydospores. 1: *pollux*; 2: *sec13* × *nup133*; 3: *nup133*; 4: *sec13*; 5: Col-0.

We investigated the influence of *A. thaliana* HCSGs on the host growth-promoting effect of *P. indica*. As a control, we included the closely related sebacinoid fungus *P. williamsii* (Basiewicz et al., 2012; Lahrmann et al., 2013), which did not induce or induced very little growth promotion of *A. thaliana* Col-0 (Lahrmann et al., 2013). Wild-type and mutant plants inoculated with *P. indica* had a significant higher mean fresh weight than control or *P. williamsii*-inoculated plants (Figure 5). Importantly, wild-type and mutant roots did not differ in their biomass upon *P. indica* inoculation. We conclude that the HCSGs *POLLUX, NUP133*, and *SEC13* are not required for the growth promotion of *A. thaliana* by *P. indica*, confirming previous observations with *atpollux* mutants (Shahollari et al., 2007).

## Conclusions

Despite the similarities between colonization of plant roots by AM fungi and *P. indica*, our data indicate that CSGs which are essential for AM development (Gutjahr and Parniske, 2013) are not required for root colonization by *P. indica*. In the AM symbiosis, signal transduction for the initiation of the intracellular accommodation program is mediated by the products of CSGs (Takeda et al., 2012). Since *P. indica* intracellular colonization was observed in the absence of individual CSGs or existing homologs in *A. thaliana*, we conclude that alternative pathways must exist that support intracellular accommodation of *P. indica*. Conceptually this could be achieved through the manipulation of general programs such as polarized secretion, endocytosis, plant immunity, and/or phytohormone signaling (Schäfer et al., 2009; Dörmann et al., 2014; Evangelisti et al., 2014). Identification of such compatibility programs is of prime interest because they might offer entry ports not only for beneficial fungi like *P. indica* but also to hyphal pathogens with similar infection strategies. This is in agreement with the recent observation that CSG mutants of *M. truncatula* show unaltered infection and haustorial development by the phytopathogenic oomycete *Phytophthora palmivora* (Rey et al., 2014). However, little is known about plant factors that are directly involved in the intracellular accommodation of *P. indica*. Tubby-like proteins, implicated in vesicle trafficking in mammals (Mukhopadhyay and Jackson, 2011), are required for normal colonization of *A. thaliana* roots by *P. indica*, and have been pinpointed as possible compatibility factors during the early stages of plant–fungus interaction (Reitz et al., 2012, 2013).

### Conflict of interest statement

The authors declare that the research was conducted in the absence of any commercial or financial relationships that could be construed as a potential conflict of interest.
