# Supplementary material for: Colonization of root cells and plant growth promotion by Piriformospora indica occurs independently of plant common symbiosis genes
Source: Front Plant Sci. 2015 Sep 17;6:667. doi: 10.3389/fpls.2015.00667 (PMC4585188; doi:10.3389/fpls.2015.00667)
Supplement: Supplementary file 5 [file Presentation1.PDF]

Supplementary Material

Colonization of root cells and plant growth promotion by *Piriformospora indica* occurs independently of plant common symbiosis genes

Aline Banhara[1], Yi Ding[2,*], Regina Kühner[1], Alga Zuccaro[3], and Martin Parniske[1]
[1] University of Munich (LMU), Faculty of Biology, Genetics, Martinsried, Germany
[2] Max-Planck-Institute for Terrestrial Microbiology, Marburg, Germany
[3] University of Cologne, Botanical Institute, Cluster of Excellence on Plant Sciences (CEPLAS), Cologne, Germany
[*] Present address: Boyce Thompson Institute for Plant Research, Ithaca, New York, U.S.A.

Correspondence: Prof. Dr. Martin Parniske, University of Munich (LMU), Faculty of Biology, Genetics, Großhaderner Strasse 4, 82152, Martinsried, Germany.
parniske@lmu.de

1. Supplementary Figures and Tables

1.1. Supplementary Tables

Supplementary Table 1. *L. japonicus* common symbiosis mutants used in this study.

Mutant	Reference	Line descriptor	Seed bag(s)
castor-12	(Imaizumi-Anraku et al., 2005)	SL3251.2	79827/79828
ccamk-13	(Perry et al., 2009)	cac57.9	86273
cyclops-3	(Szczyglowski et al., 1998)	EMS126	78527/78602
nup85-1	(Szczyglowski et al., 1998)	EMS76	88351
nup133-1	(Schauser et al., 1998)	5371-22	88429/88430
pollux-5	(Imaizumi-Anraku et al., 2005)	SL3130	86523/86524
symrk-3	(Stracke et al., 2002)	cac41.5	86966/86967

Supplementary Table 2. *A. thaliana* HCSG mutants used in this study

Gene/Ecotype	Gene ID	NASC ID	Seed bags
POLLUX	At5g49960	N566135	435/437
SEC13	At3g01340	N662322	190/283
NUP133	At2g05120	N565761	197/333
SEC13 x *NUP133*			577/580
ShRK1 x *ShRK2*	At1g62270/At2g37050	N643700/ N467036	1906/1907

1.2. Supplementary Figures

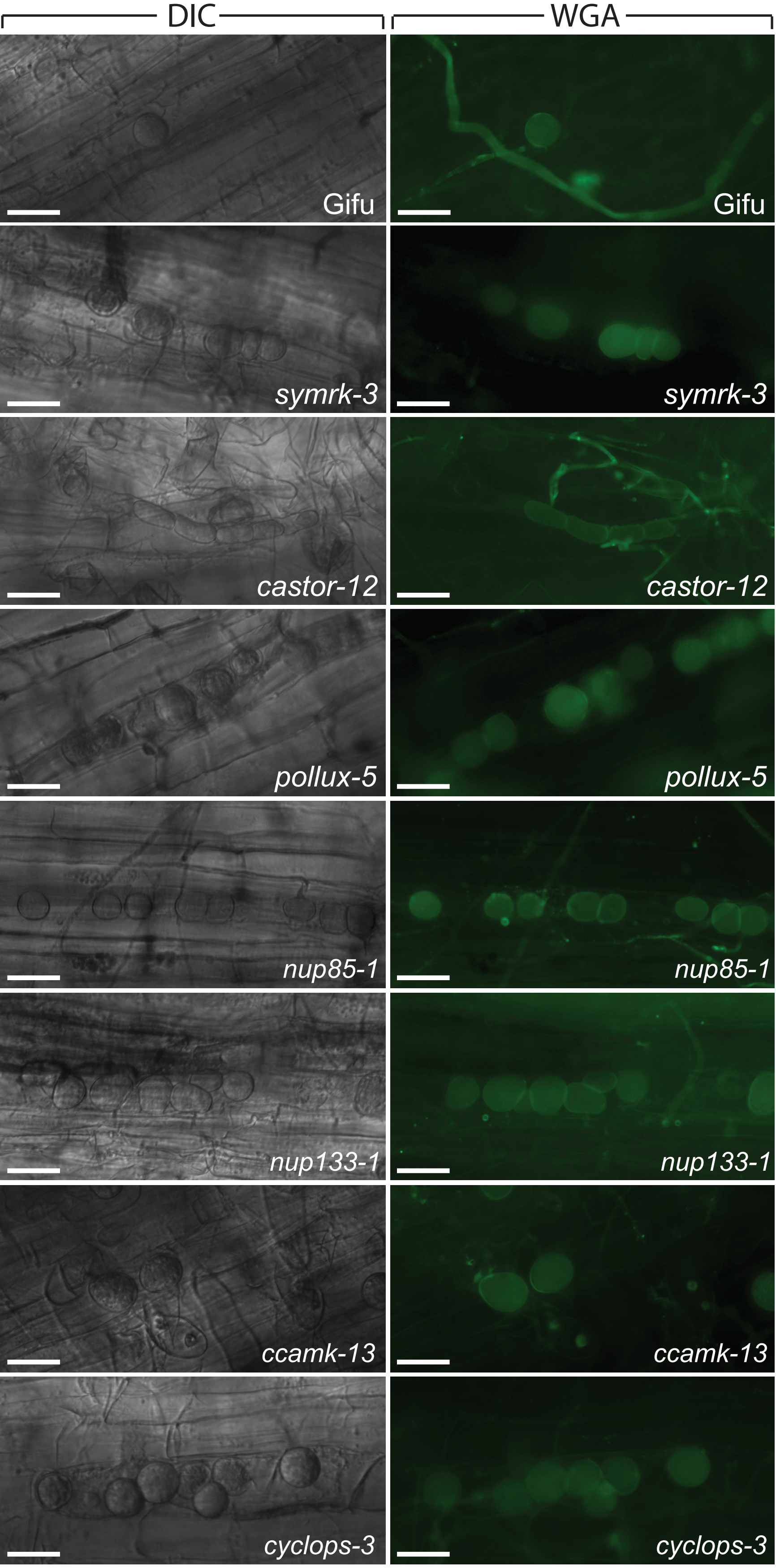

Supplementary Figure 1. *P. indica* sporulation inside root cells of *L. japonicus*. Single and multiple spores are visible inside individual cleared root cells or root hairs of *L. japonicus* wild-type (Gifu) and in the indicated common symbiosis mutants at 14 dpi. Left column: differential interference contrast (DIC) microscopy; right column: WGA-AF488 (WGA) detected by fluorescence microscopy. Scale bar: 25 µm.

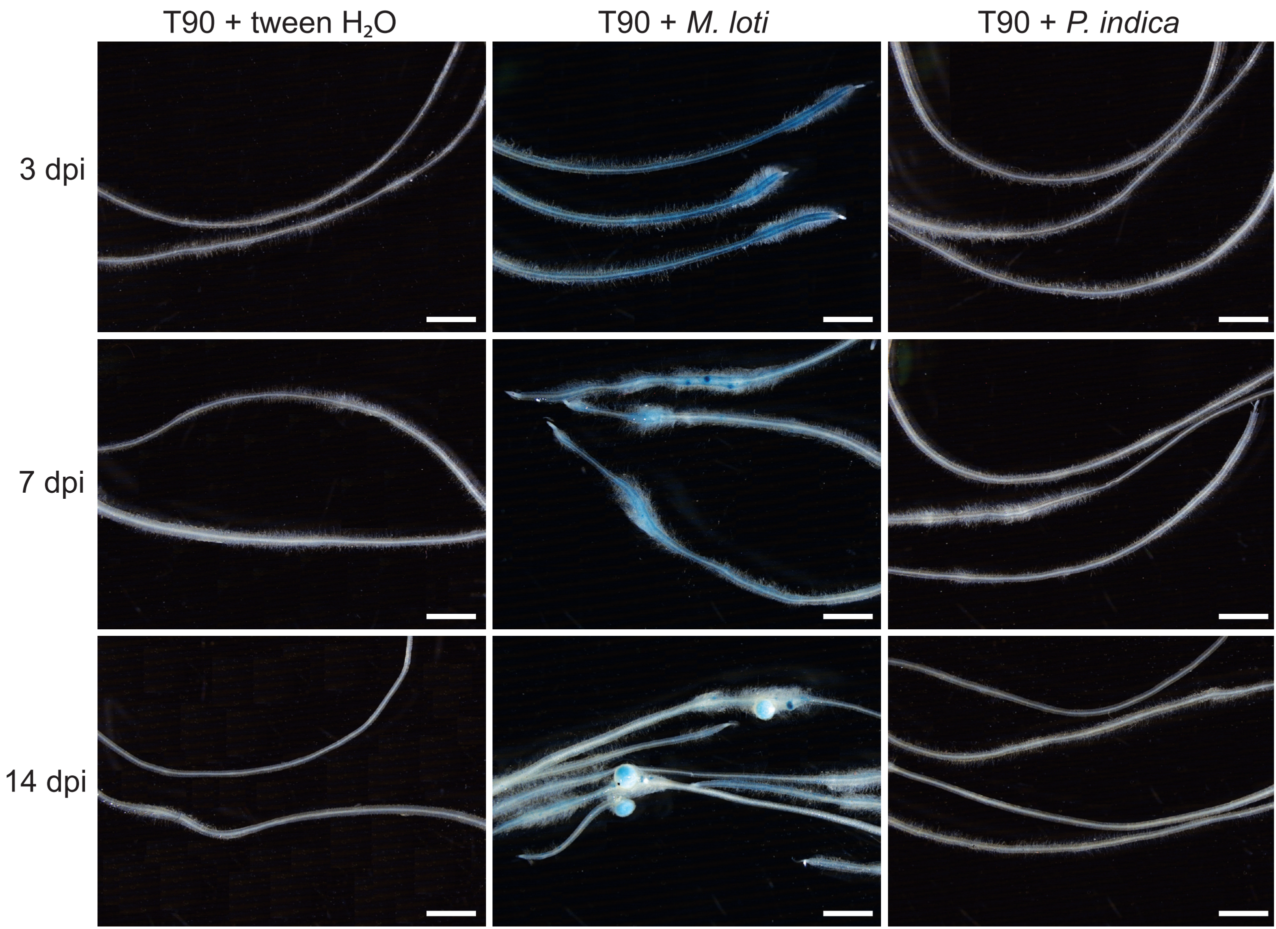

Supplementary Figure 2. No GUS expression in the *L. japonicus* symbiosis reporter line T90 inoculated with *P. indica*. At all evaluated time-points, GUS activity was not observed in T90 plants inoculated with *P. indica* chlamydospores or mock-inoculated with Tween water. *Mesorhizobium loti Ds*Red was used as a positive control and activated GUS expression (blue) in the root or root nodules at all time-points analyzed. Scale bar: 2.5 mm.

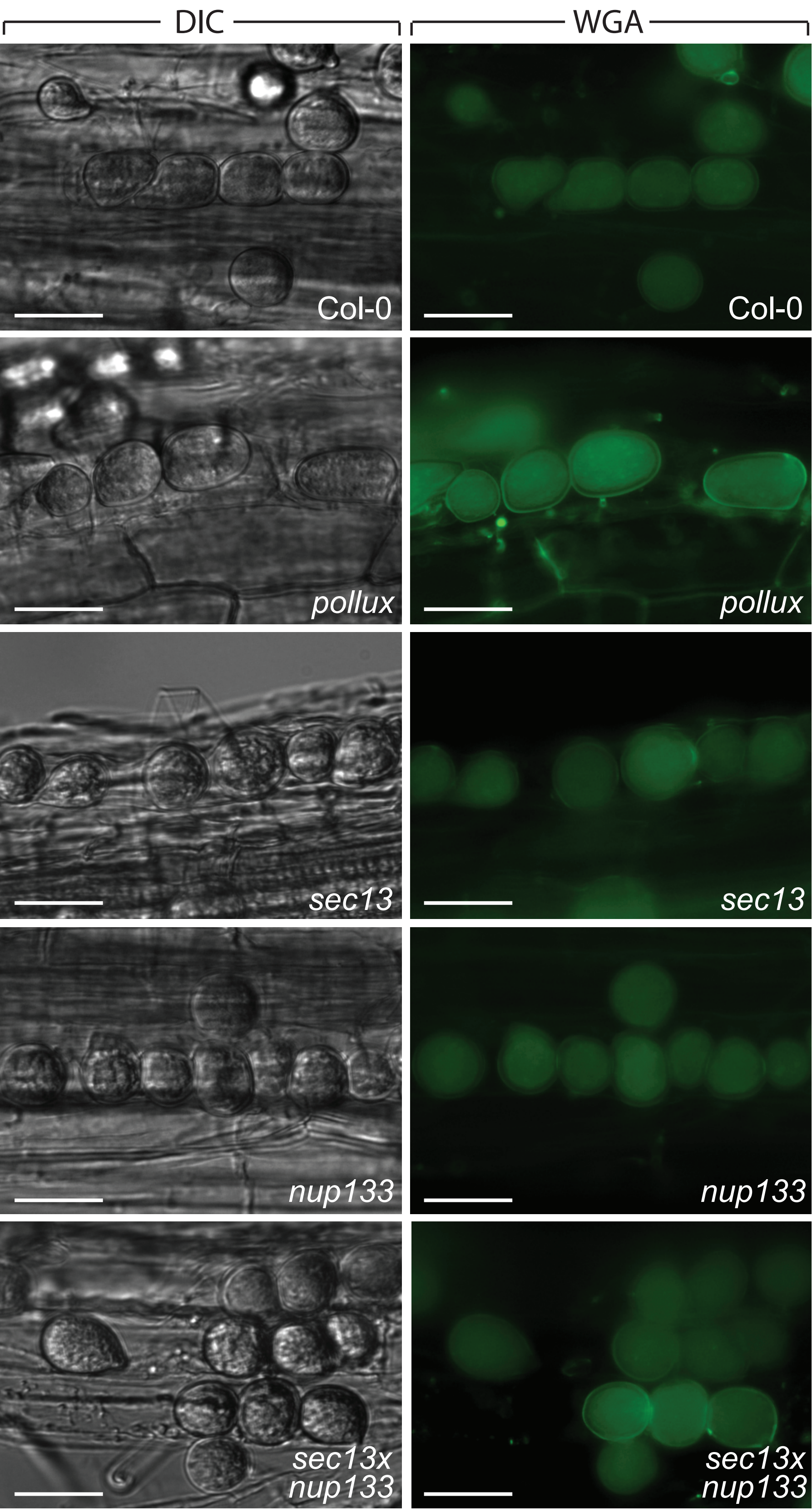

Supplementary Figure 3. Intracellular sporulation of *P. indica* in *A. thaliana* roots. Multiple spores are present within individual root cells in *A. thaliana* wild-type (Col-0) as well as in the indicated HCSG mutants at 14 dpi. Left column: differential interference contrast (DIC) microscopy. Right column: WGA-AF488 (WGA) detected by fluorescence microscopy. Scale bar: 25 µm.

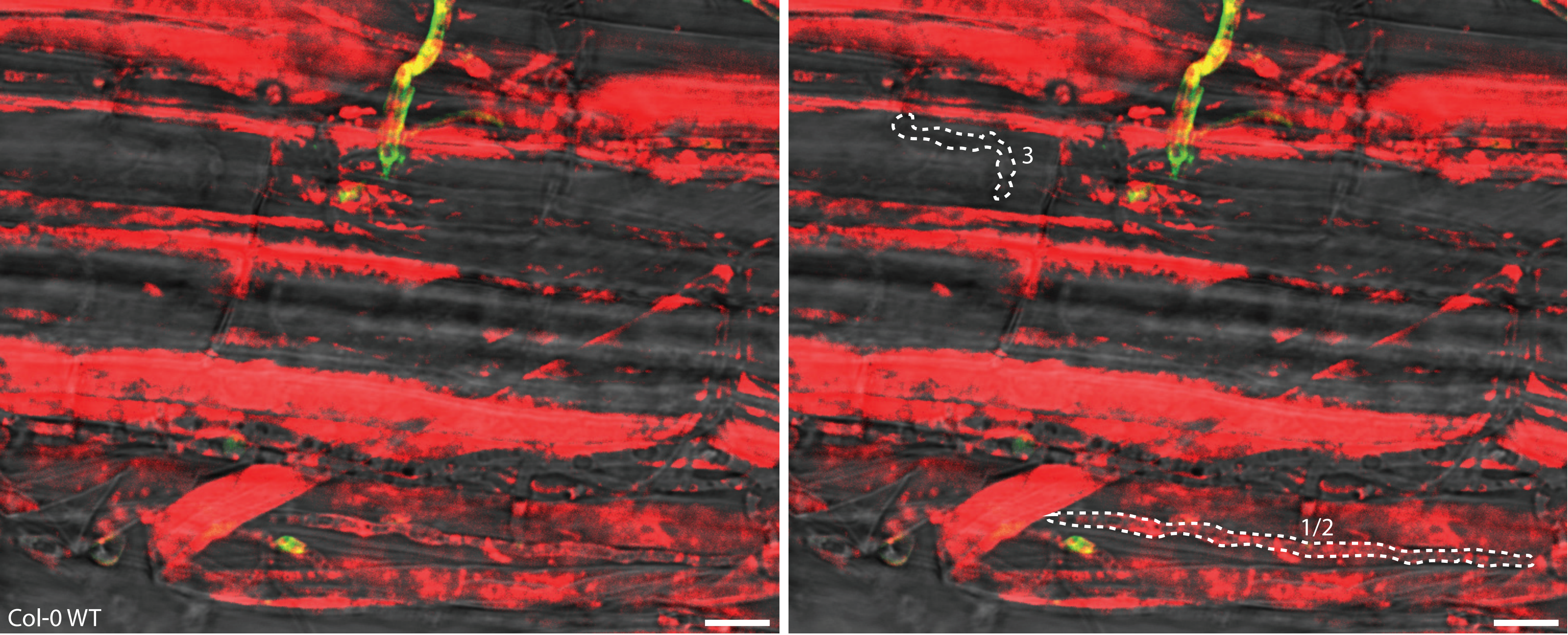

Supplementary Figure 4. Stages of *A. thaliana* root colonization by *P. indica*. Intracellular hyphae are outlined by dotted lines on the right side, and numbers indicate stages of *P. indica* root colonization. An intracellular hyphae occurs within a root cell that is filled with FM4-64-stained structures, indicating stage 1 or 2 of root colonization (labeled "1/2"), while another hyphae is shown within a cell lacking stained contents (labeled "3"). Extracellular hyphae are stained with WGA-AF488 (green), whereas intracellular hyphae are not or weakly fluorescent. Images were acquired at 4 dpi with *P. indica* chlamydospores. Scale bar: 15 µM.

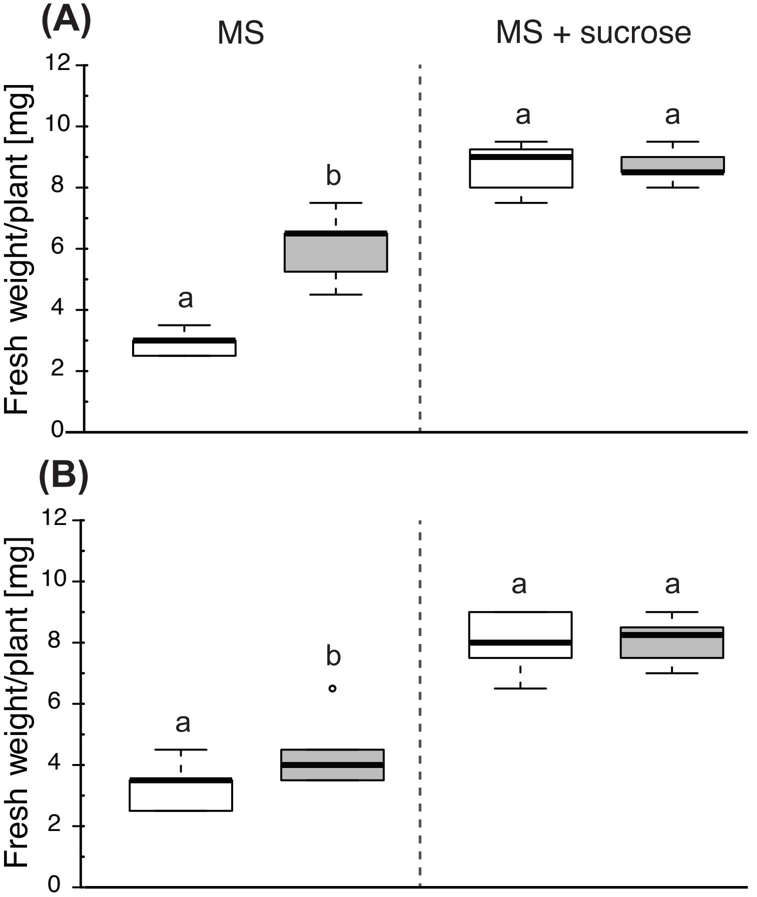

Supplementary Figure 5. Effect of sucrose on the growth promotion of *A. thaliana* plants by *P. indica*. Box plots represent the fresh weight of Col-0 plants (ca. 15/treatment) grown on media with or without sucrose, 7 dpi with Tween water or *P. indica* chlamydospores. Grey boxes: *P. indica*–inoculated, white boxes: mock-inoculated. Without sucrose addition, the mean fresh weight of *P. indica*-inoculated plants was significantly higher than that of mock-inoculated plants ($p < 0.05$). In the presence of sucrose, the mean fresh weight of the plants grown in the presence of *P. indica* was not significantly different from that of mock-inoculated plants ($p > 0.05$). Statistical analyses were performed with a Kruskal-Wallis test followed by a Bonferroni-Holm correction using the mock-inoculated samples as control group. Plots sharing a letter do not significantly differ (significance level: 5%). Open circles: outliers. Results from two independent experiments (A and B) are shown.

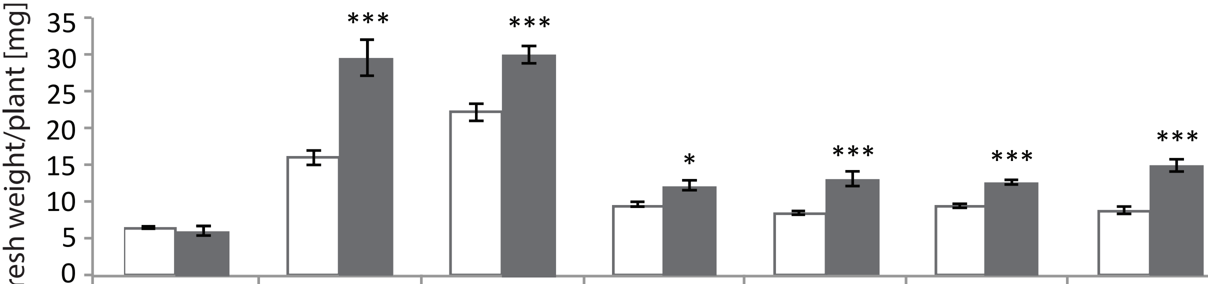

Salt (mM)	0 NO_3	0.5 NO_3	5 NO_3	10 NH_4Cl	20 NH_4Cl	0 PO_4	modified HO (standard)
KNO_3	0	0.5	5	5	5	5	5
$Ca(NO_3)_2$	0	0	0	5	5	5	5
KH_2PO_4	4	4	4	4	4	0	4
NH_4Cl	0	0	0	10	20	0	0

Supplementary Figure 6. Influence of $Ca(NO_3)_2$, KNO_3, KH_2PO_4 or NH_4Cl concentrations on the growth promotion of *A. thaliana* by *P. indica*. Bars represent the fresh weight of *P. indica*-inoculated plants (ca. 10 plants/treatment) relative to the respective mock-inoculated at 7 dpi, and error bars refer to the standard deviation of the mean of the fresh weight values. Overall, co-cultivation with *P. indica* chlamydospores had a positive effect on the mean fresh weight of the plants, independent of nutrient concentration, except for medium depleted of any nitrogen source ($p > 0.05$). Plants were grown on the modified HO medium with the indicated salt concentrations. White box: mock-inoculated; grey box: *P. indica*-inoculated. Statistical analyses were performed with a Kruskal-Wallis test followed by a Bonferroni-Holm correction using the mock-inoculated samples as control group, and P was calculated on the absolute values. Each pair mock/*P. indica*-inoculated was compared individually. Stars indicate a significant difference (at the 5% significance level) relative to the mock-inoculated control. *, $P < 0.05$; ***, $P < 0.001$.

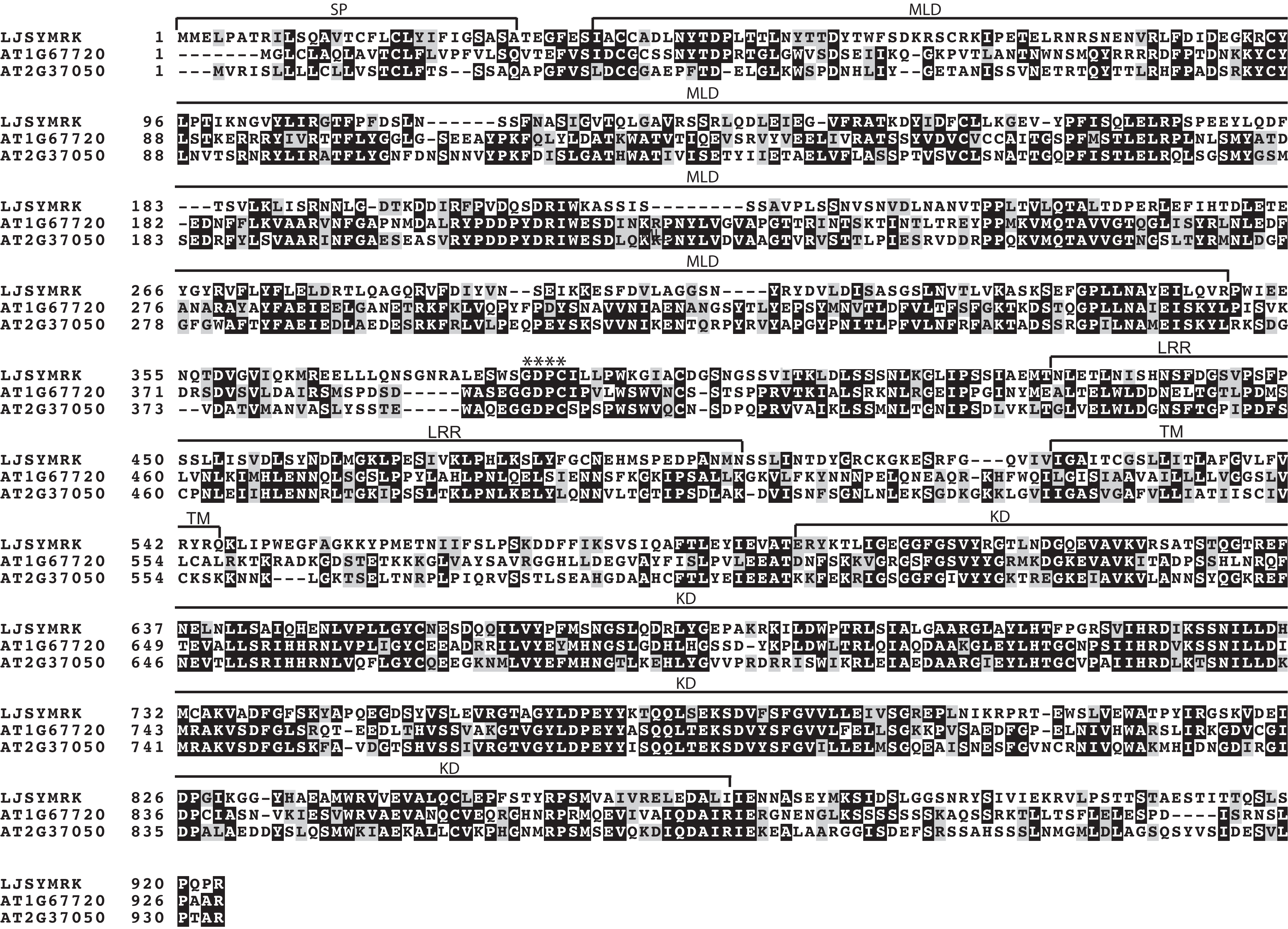

Supplementary Figure 7. Alignment between the protein sequences of *L. japonicus SYMRK* (accession number AAM67418) and *A. thaliana ShRK1* (AT1G67720) and *ShRK2* (AT2G37050). Identical and conserved amino acid residues are shown in black and in grey, respectively. The depicted domains were identified using ScanProsite (de Castro et al., 2006) and InterProScan (Jones et al., 2014), as well as data available from Markmann et al. (2008). SP, signal peptide; MLD, malectin-like domain; LRRs, leucine-rich repeats; TM, transmembrane domain; KD, kinase domain.

```
                                     1B          1C          1D
LJSEC13-like1    1  --MPAQKVETGHQDTVHDVAMDYYGKRLATASSDHTIKIIGVSNT-ASQHLAT---LAGH
LJSEC13-like2    1  --MPGQKVETGHQDTVHDVAMDYYGKRLATASSDHTIKIIGVSIA-ASQHLAT---LTGH
AT3G01340        1  --MPPQKIETGHSDTIHDVVMDYYGKRVATASSDCTIKITGVSNSGGSQHLAT---LTGH
AT2G30050        1  --MPGQKIETGHEDIVHDVQMDYYGKRIATASSDCTIKITGVSNNGGSQQLAT---LTGH
yeastSEC13       1  ----MVVIANAHNELIHDAVLDYYGKRLATCSSDKTIKIFEVEGE-THKLIDT---LTGH
humanSEC13       1  MVSVINTVDTSHEDMIHDAQMDYYGTRLATCSSDRSVKIFDVRNG-GQILIAD---LRGH
LJNENA           1  ----MAKEVLTLDNGTTCSSWNYSGTRLAAGSADGTLSIFDSRDPPSSSSLRLSFKSKVH
ATSEH1           1  ----MAKSMATLDSGTTCSSWNQSGDRLAAGSLNGKLSIYES-STSSSSTFSCTSKVRVS
yeastSEH1        1  ----MQPFDSGHDDLVHDVVYDFYGRHVATCSSDQHIKVFKLDKDTSNWELSDS--WRAH
humanSEH1        1  -MFVARSIAADHKDLIHDVSFDFHGRRMATCSSDQSVKVWDKS-ESGDWHCTAS--WKTH

                         2A        2B        2C                     2D
LJSEC13-like1   55  QGPVWQVAWAHPKFGSMIASCSYDGRVIIWKEGNQ---------NEWIQAHVFDDHKSSV
LJSEC13-like2   55  QGPVWQVAWAHPKFGSLLASCSYDGRVILWKEGNQ---------NEWTQAHVFDEHKSSV
AT3G01340       56  RGPVWQVAWAHPKFGSLLASCSYDGQIILWKEGNQ---------NQWTQAHVFTDHKVSV
AT2G30050       56  RGPVWEVAWAHPKYGSILASCSYDGQVILWKEGNQ---------NQWTQDHVFTDHKSSV
yeastSEC13      53  EGPVWRVDWAHPKFGTILASCSYDGKVLIWKEEN----------GRWSQIAVHAVHSASV
humanSEC13      57  EGPVWQVAWAHPMYGNILASCSYDRKVIIWREEN----------GTWEKSHEHAGHDSSV
LJNENA          57  EGNIVNIVWVPPAYGDAVACISVDGIVSLLEEVVEDSQP-----LQWKICKCFGSSSDKV
ATSEH1          56  ESSIVKIVWLPSEYGDAVACVCEDGSLSIWEELSEDAHG-----LEWKLCKSMKNKSSQV
yeastSEH1       55  DSSIVAIDWASPEYGRIIASASYDKTVKLWEEDPDQEECSGR---RWNKLCTLNDSKGSL
humanSEH1       57  SGSVWRVTWAHPEFGQVLASCSFDRTAAVWEEIVGESNDKLRGQSHWVKRTTLVDSRTSV

                      3A          3B         3C          3D            4A
LJSEC13-like1  106  NSVVWAPHELGLCLACGSSDGNISVFTARADGGWDTSRIDQAHPVGVTSVSWAPSM---A
LJSEC13-like2  106  NSVAWAPHELGLCLACGSSDGNISVFTARADGGWDTSRIDQAHPVGVTSVSWAPST---A
AT3G01340      107  NSIAWAPHELGLSLACGASDGNISVFSARADGGWDTTKIDQAHPVGVTSVSWAPAT---E
AT2G30050      107  NSIAWAPHDIGLSLACGSSDGNISVFTARADGGWDTSRIDQAHPVGVTSVSWAPAT---A
yeastSEC13     103  NSVQWAPHEYGPLLLVASSDGKVSVVEFK-ENGTTSPIIIDAHAIGVNSASWAPAT---I
humanSEC13     107  NSVCWAPHDYGLILACGSSDGAISLLTYTGEGQWEVKKINNAHTIGCNAVSWAPAV---V
LJNENA         112  LDVQFGISLASLKMVAAYSDGHMKVFELLDPLELKNWQLQAEFQNVIESVSSFGKAVCLS
ATSEH1         111  LDVQFGVSRKSLKMVAAYSDGYLRVFELLNPLELKNWQLQAEFQNVIDSLSTLGKPSSLS
yeastSEH1      112  YSVKFAPAHLGLKLACLGNDGILRLYDALEPSDLRSWTLTSEMKVLSIPPANHLQS---D
humanSEH1      117  TDVKFAPKHMGLMLATCSADGIVRIYEAPDVMNLSQWSLQHEISCKLSCSCISWNP---S

                                           4B          4C       4D
LJSEC13-like1  163  PGALVG--AG----LLDPVQKLCSGGCDNTVKVWKLT-NGNWKMDCFPALHMHT--DWVR
LJSEC13-like2  163  PGALVS--GG----LLDPVQKLCSGGCDNTVKVWKLN-NGLWKMDCFPALQMHT--DWVR
AT3G01340      164  PGALVS--SG----MIDPVYKLASGGCDSTVKVWKFS-NGSWKMDCFPALNKHT--DWVR
AT2G30050      164  PGALVS--SG----LLDPVYKLASGGCDNTVKVWKLA-NGSWKMDCFPALQKHT--DWVR
yeastSEC13     159  EEDGEH--NG-----TKESRKFVTGGADNLVKIWKYN-SDAQTYVLESTLEGHS--DWVR
humanSEC13     164  PGSLIDHPSGQ---KPNYIKRFASGGCDNLIKLWKEEEDGQWKEE--QKLEAHS--DWVR
LJNENA         172  ASISFNPQNGGSQESSFLVGFNSNTSELNSAKVWEFDQAHQRWLPVAELAVPEDKGDQVY
ATSEH1         171  ASVSWNPMKGEEQEPSFVLAFNSDSPHLNSSKIWEFDEAHNRWLAVAELALPEDKGDPVY
yeastSEH1      169  FCLSWCP-------SRFS-PEKLAVSALEQAIIYQRG-KDG-KLHVAAKLPGHK--SLIR
humanSEH1      174  SSRAHSP-------MIAVGSDDSSPNAMAKVQIFEYN-ENTRKYAKAETLMTVT--DPVH

                      5A                  5B       5C
LJSEC13-like1  214  DVAWAPNLGLPKSTIASASQDGKVIIWTVGKEGD-QWEGKILN-----------------
LJSEC13-like2  214  DVAWAPNLGLPKSTIASASQDGKVIIWTVAKEGD-QWEGRVLN-----------------
AT3G01340      215  DVAWAPNLGLPKSTIASGSEDGKVIIWTIGKEGE-QWEGTVLK-----------------
AT2G30050      215  DVAWAPNLGLPKSTIASGSQDGKVIIWTVGKEGE-QWEGKVLK-----------------
yeastSEC13     209  DVAWSPTV-LLRSYLASVSQDRTCIIWTQDNEQG-PWKKTLLKE----------------
humanSEC13     217  DVAWAPSIGLPTSTIASCSQDGRVFIWTCDDASSNTWSPKLLH-----------------
LJNENA         232  AVAWAPNIGRTYEIIAVATHKG-LAIWHLGLNPDHDGRLPVER-----------------
ATSEH1         231  ALSWAPNIGRPYEVVAVATHKG-IGIWHVGLAPDLEGRLPVKK-----------------
yeastSEH1      217  SISWAPSIGRWYQLIATGCKDGRIRIFKITEKLSPLASEESLTNSNMFDNSADVDMDAQG
humanSEH1      224  DIAFAPNLGRSFHILAIATKD--VRIFTLKPVRKELTSSGGPT-----------------

                                                       6A          6B       6C
LJSEC13-like1  256  -------------------------DFKTPVWRVSWSLTGNILAVADGNNNVTLWKEAVD
LJSEC13-like2  256  -------------------------DFKTPVWRASWSLTGNILAVADGNNNVTLWKEAVD
AT3G01340      257  -------------------------DFKTPVWRVSWSLTGNLLAVSDGNNNVTVWKESVD
AT2G30050      257  -------------------------DFMTPVWRVSWSLTGNLLAVSDGNNNVTVWKEAVD
yeastSEC13     251  ------------------------EKFPDVLWRASWSLSGNVLALSGGDNKVTLWKENLE
humanSEC13     260  -------------------------KFNDVVWHVSWSITANILAVSGGDNKVTLWKESVD
LJNENA         274  --------------------VAVLSGHDGMVWQMEWDMSGMTLATTGQDGMVRLWQSNLN
ATSEH1         273  --------------------VSSLSGHQGEVWQMEWDMSGMTLASTGSDGMVKLWQSNLN
yeastSEH1      277  RSDSNTEEKAELQSNLQVELLSEHDDHNGEVWSVSWNLTGTILSSAGDDGKVRLWKATYS
humanSEH1      265  --------------KFEIHIVAQFDNHNSQVWRVSWNITGTVLASSGDDGCVRLWKANYM

LJSEC13-like1  291  GEWQQVTTVDP---------------------------------------
LJSEC13-like2  291  GDWQQVTTVEP---------------------------------------
AT3G01340      292  GEWEQVTVVEP---------------------------------------
AT2G30050      292  GEWEQVTAVEP---------------------------------------
yeastSEC13     287  GKWEPAGEVHQ---------------------------------------
humanSEC13     295  GQWVCISDVNKGQGSVSASVTEGQQNEQ----------------------
LJNENA         314  GVWHQQAAFEPTS-------------------------------------
ATSEH1         313  GEWHEQATLEPVPS------------------------------------
yeastSEH1      337  NEFKCMSVITAQQ-------------------------------------
humanSEH1      311  DNWKCTGILKGNGSPVNGSSQQGTSNPSLGSTIPSLQNSLNGSSAGRKHS
```

Supplementary Figure 8. Alignment between SEC13-related proteins in *L. japonicus*, *A. thaliana*, yeast and human. Alignment was performed between the protein sequences encoded by *LjSEC13-like1* (accession number AB506697), *LjSEC13-like2* (accession number AB506698), *A. thaliana SEC13* candidates (At3g01340 and At2g30050), yeast *SEC13* (accession number Q04491), human *SEC13* (accession number P55735), *LjNENA* (AB506696), *AtSEH1* (At1g64350), yeast *SEH1* (accession number P53011), and human *SEH1* (accession number A8K5B1). Identical amino acid residues are shown in black, and conserved amino acids in grey. Predicted WD-40 domains are highlighted by black lines above the alignment. Putative β-propeller blades (indicated by arrows) are based on predictions by Groth et al. (2010) for NENA, the *L. japonicus* SEH1 (SEC13 Homolog). Labels refer to blade number and strand order.

```
                                                                                         7D        α1
LJNUP133    1 MFSCGTKKNNGASRDQARASS---VLDSPVTPP--LRSSAVPNRPPTGTPAPWTPRLSVLARVPQVNRTGKEDNTDPIKPVFVAEFPQVVRDEQA
ATNUP133    1 MFSPLTKRAKQSSRNEKTPRNRVPPPDSPVTPATQNRNNFISDRPATGTPAPWAPRLSVLARVSPGNNGDKGVDSDQLKPVFVGEFPQLLRDEQS
                       1A            1B        1C            1D                            2A
LJNUP133   91 ALLHKRVPVEGCASGGIDKSTSLAWIISGNRVFIWSYLSPASSMKCVVLEIP-----FNDGDVGKHEAGSWLLCVVNYDGASSGANKVAKHCNSA
ATNUP133   96 ------YPGDACVSGGMDKETCLSWFITGSKVFVWSHLTTLPSRKCVVLELPVVVLVNEESGSGLQDGKSWLVNVVSWDTSAGAATRASRSRSPV
               2B       2C                       2D                          3A          3B          3C
LJNUP133  181 AVVLCNRKTRAVVYWPDIYSQSRNAPVTNLVSSDEFVTSLVSDGKTFSNWLRRPSSLNELHMFNSMIASAVPGCDFVCVAFACSSSGKLWQFKCS
ATNUP133  185 GVVMCNRKTRAVVYWSDIFSGQEAAPAEKAR-------HLIKRQ---SNGIR--SSRAENSDLNSLITTAVAAAERLCIAIACSSNGELWQFTCS
                   3D                           4A          4B          4C                4D
LJNUP133  276 PSGIHRTEVCESSMHLPPEGGDSGQLEGNKGYPRSLTWCFPHHSTKESNRQFLVLTDHEIKCFGVEFSSDIHVSMLWSQVIVGTDPELGIKKDLA
ATNUP133  268 PTGVKSNQVQLN----------ISSSSVSEGYPRSLIWRFSQGLARESCWEFLMLTDCDIHCFTIEPYPDLTVSEVWQHEIVGTDGDSGIKKDIA
                     5A          5B                                                  5D           6A
LJNUP133  371 GQKGIWPLDVQVDDYGKVITILFATFCKDRISSSSYMQYSLLTMQYKSGLDVG-TTNDKILEKKAPIEVIIPKARVEAEDFLFSMRLRIGGKPSG
ATNUP133  353 SQKQIWPLDLQVDDQGKVITVLVATICMDRASSSSYTQYSLLTLQHKSEMRFADGREEKVLEKQGPIQVIIPKARVEDKDFLFSMRLRVGGRPPG
               6B       6C       6D          7A              7B      7C
LJNUP133  465 STVIISGDGTATVSHYYRNATRLYQFDLPYDAGKVLDASILPSADDHAEGAWVVLTEKAGMWAIPEKAVILGGVEPPERSLSRKGSSNERSAQEE
ATNUP133  448 SAIILSGDGTATVCYCHGSSTRLYKFDLPYDAGKVLDASVLSSTDEHEYGAWTVLTEKAGVWAIPEKAVVLGGVEPPERSLSRKNSSNERSTRDE

LJNUP133  560 IRNLTFAGNFAPRRASSEAWGTGDRQRAVLSGIARRTAQDEESEALLNQLFNEFLSSGQVDRSLEKLETSGSFERDGEINVFVRMSKSIIDTLAK
ATNUP133  543 TRVTPYGVDRTAGRENSDIQNIEDKGNPKMG-FTRQTARDEESEALLGQLFEGFLLSGKVDGSLEKLSQSGAFDRDGEANVFARKSKSIVDTLAK

LJNUP133  655 HWTTTRGAEILAMAYVSTQLLEKQQKHQKFLHFLALSKCHEELCSRQRHALQLILEHGEKLSAMIQLRELQNLISQNRSTGVGSSNSSLDIQLAG
ATNUP133  637 HWTTTRGAEIVAMTVISSQLVEKQQKHENFLHFLALSKCHEELCSKQRHSLQIILENGEKLAAMIQLRELQNMINQNRSARFGSPQAGSEDQVSC

LJNUP133  750 ALWDMIQLVGDRARRNTVLLMDRDNAEVFYSKVSDLENFFYCLDAELEYVIRPEHPSGIQIQRTCELSNACVTIIRTCFDYKNENQLWYPPPEGL
ATNUP133  732 ALWDLIQFVGERARRNTVLLMDRDNAEVFYSKVSELEEVFYCLNRQLEYIIRADQPLGTQLQRACELSNACVTILQTALDYKNEHQMWYPPLEGL

LJNUP133  845 TPWYCQPAVRKGIWSVASVLLQLLNETSGLDKTAKLNLYNHLEAVAEVLLEAYSGAVTAKIEREEEHKGLLDEYWKRRDALLETLHQQIKEFEAT
ATNUP133  827 IPWHSQTVVCNGLWCIASFMLHLLTEASRIDISAKSDIYTHLEVLTEVLLEACAGSTFAKLEREEENKGLLNEYWTRRDTIFDSLYRQAKEFMEA

LJNUP133  940 HKDSIEGAEEQNDEAIMK-LTSRLLSIAKQHGCYKVMWTVCCDVNDSELLRNIMRESLGPDGGFSYYVFEKLHENRQFSELLKLGEEFPEELSIF
ATNUP133  922 EIQGIRERTEATDEDIFRNRCSNLISIAKRHAGYKIMWKICYDLNDTGLLRNLMHEGVGPQGGFSYFVFQQLYDMKQFSKLLRLGEEFQDELLIF

LJNUP133 1222 KNTVLFQASSRCYGP-RSESFEEGFDQVLPLRQENMETSMLGDMSSSVETILMQHKDFPVAGKLMLMAVMLGSDHDGDIGVEEGPSPMEKILKLQ
ATNUP133 1202 RNTALFQASKRCYGPTRVNTFDGDFAQVLPLRRENPE-----DSTSSVEDVLMSHKDFAEAGKLMLTAIMLGCVEEEGIVAEEFSSPMENLKKLQ

LJNUP133 1127 EEVMKRLPSVEDKQLIEDQLLHPEDLIKLCLEGEDGELSLWTFDVFAWTSSSFRKTHRKLLEDCWKKASSQDDWSKFHDSCMVEGWSDEETLQNL
ATNUP133 1111 EEITKALPNGEAR----NRLFRPEELIETCLNIQGRWTAIKAFEVFAWTSSSFRENHRSLLEECWRNAADQDDWDRHHQASTNEGWSEEETLQNL
```

Supplementary Figure 9. Alignment between the protein sequences of *NUP133* candidate orthologs in *L. japonicus* (accession number CAI64810) and *A. thaliana* (At2g05120). Identical amino acid residues are shown in black, and conserved residues in grey. LJNUP133 was predicted to have an N-terminal α/β domain and C-terminal α helical domain, localized between residues 50–535 and 555-1309, respectively (Kanamori et al., 2006). Putative β-propeller blades (in boxes) contain individual β strands (arrows) labelled according to number and strand order, as predicted by Kanamori et al. (2006) based on the human NUP133 sequence (Berke et al., 2004). The first α-helix domain is shown here based on domain prediction by Berke et al. (2004) and an alignment between LjNUP133 and the human sequence by Kanamori et al. (2006).

1.3. Supplementary Movies

When not stated otherwise, movies show roots stained with WGA-AF488 (green) to visualize fungal structures and FM4-64 (red) for membranes, and represent approximately 60 s with 11 frames of ca. 5.5 s each. The observed movement of FM4-64 stained cellular content was taken as evidence for cell activity

Supplementary Movie 1: *A. thaliana* wild-type (Col-0) roots showing stage 1 of colonization by *P. indica*. Intracellular movement appears similar between a colonized and neighboring non-colonized cells. A non-stained intracellular hypha is indicated by a white arrowhead.

Supplementary Movie 2: *A. thaliana* wild-type (Col-0) roots showing stage 2 of colonization by *P. indica*. Intracellular movement in fungus-free cells is higher than in colonized cells, where immobile structures are present next to moving vesicles.

Supplementary Movie 3: Non-colonized *A. thaliana* Col-0 root showing 3 types of cells according to their viability. Roots were stained with FM4-64. Movie represents a time lapse of 60 s (18 frames of ca. 3.3 s each). Intracellular movement can be observed in the majority of the cells, indicative of activity, while accumulation of cellular debris is indicated (white arrow) in a cell that is likely dying. Non-fluorescent cells are present in the center of the root. These cells appear devoid of cytoplasm, and are therefore probably dead.

Supplementary Movie 4: Stage 1 of *P. indica* biotrophy in roots of the *A. thaliana* HCSG mutant *pollux*. Intracellular movement appears similar compared to a wild-type cell at the same colonization stage. Non-stained intracellular hyphae are indicated by white arrowheads.

2. References

Berke, I.C., Boehmer, T., Blobel, G., and Schwartz, T.U. (2004). Structural and functional analysis of Nup133 domains reveals modular building blocks of the nuclear pore complex. *J Cell Biol* 167, 591-597. doi: 10.1083/jcb.200408109.

De Castro, E., Sigrist, C.J., Gattiker, A., Bulliard, V., Langendijk-Genevaux, P.S., Gasteiger, E., Bairoch, A., and Hulo, N. (2006). ScanProsite: detection of PROSITE signature matches and ProRule-associated functional and structural residues in proteins. *Nucleic Acids Res* 34, W362-365. doi: 10.1093/nar/gkl124.

Demchenko, K., Winzer, T., Stougaard, J., Parniske, M., and Pawlowski, K. (2004). Distinct roles of *Lotus japonicus* SYMRK and SYM15 in root colonization and arbuscule formation. *New Phytologist* 163, 381-392. doi: 10.1111/j.1469-8137.2004.01123.x.

Groth, M., Takeda, N., Perry, J., Uchida, H., Draxl, S., Brachmann, A., Sato, S., Tabata, S., Kawaguchi, M., Wang, T.L., and Parniske, M. (2010). *NENA*, a *Lotus japonicus* homolog of *Sec13*, is required for rhizodermal infection by arbuscular mycorrhiza fungi and rhizobia but dispensable for cortical endosymbiotic development. *Plant Cell* 22, 2509-2526. doi: 10.1105/tpc.109.069807.

Imaizumi-Anraku, H., Takeda, N., Charpentier, M., Perry, J., Miwa, H., Umehara, Y., Kouchi, H., Murakami, Y., Mulder, L., Vickers, K., Pike, J., Downie, J.A., Wang, T., Sato, S., Asamizu, E., Tabata, S., Yoshikawa, M., Murooka, Y., Wu, G.J., Kawaguchi, M., Kawasaki, S., Parniske, M., and Hayashi, M. (2005). Plastid proteins crucial for symbiotic fungal and bacterial entry into plant roots. *Nature* 433, 527-531. doi: 10.1038/nature03237.

Jones, P., Binns, D., Chang, H.Y., Fraser, M., Li, W., Mcanulla, C., Mcwilliam, H., Maslen, J., Mitchell, A., Nuka, G., Pesseat, S., Quinn, A.F., Sangrador-Vegas, A., Scheremetjew, M., Yong, S.Y., Lopez, R., and Hunter, S. (2014). InterProScan 5: genome-scale protein function classification. *Bioinformatics* 30, 1236-1240. doi: 10.1093/bioinformatics/btu031.

Kanamori, N., Madsen, L.H., Radutoiu, S., Frantescu, M., Quistgaard, E.M., Miwa, H., Downie, J.A., James, E.K., Felle, H.H., Haaning, L.L., Jensen, T.H., Sato, S., Nakamura, Y., Tabata, S., Sandal, N., and Stougaard, J. (2006). A nucleoporin is required for induction of Ca^{2+} spiking in legume nodule development and essential for rhizobial and fungal symbiosis. *Proc Natl Acad Sci U S A* 103, 359-364. doi: 10.1073/pnas.0508883103.

Kistner, C., Winzer, T., Pitzschke, A., Mulder, L., Sato, S., Kaneko, T., Tabata, S., Sandal, N., Stougaard, J., Webb, K.J., Szczyglowski, K., and Parniske, M. (2005). Seven *Lotus japonicus* genes required for transcriptional reprogramming of the root during fungal and bacterial symbiosis. *Plant Cell* 17, 2217-2229. doi: 10.1105/tpc.105.032714.

Markmann, K., Giczey, G., and Parniske, M. (2008). Functional adaptation of a plant receptor-kinase paved the way for the evolution of intracellular root symbioses with bacteria. *PLoS Biol* 6, e68. doi: 10.1371/journal.pbio.0060068.

Perry, J., Brachmann, A., Welham, T., Binder, A., Charpentier, M., Groth, M., Haage, K., Markmann, K., Wang, T.L., and Parniske, M. (2009). TILLING in *Lotus*

japonicus identified large allelic series for symbiosis genes and revealed a bias in functionally defective ethyl methanesulfonate alleles toward glycine replacements. *Plant Physiol* 151**,** 1281-1291. doi: 10.1104/pp.109.142190.

Saito, K., Yoshikawa, M., Yano, K., Miwa, H., Uchida, H., Asamizu, E., Sato, S., Tabata, S., Imaizumi-Anraku, H., Umehara, Y., Kouchi, H., Murooka, Y., Szczyglowski, K., Downie, J.A., Parniske, M., Hayashi, M., and Kawaguchi, M. (2007). NUCLEOPORIN85 is required for calcium spiking, fungal and bacterial symbioses, and seed production in *Lotus japonicus*. *Plant Cell* 19**,** 610-624. doi: 10.1105/tpc.106.046938.

Schauser, L., Handberg, K., Sandal, N., Stiller, J., Thykjaer, T., Pajuelo, E., Nielsen, A., and Stougaard, J. (1998). Symbiotic mutants deficient in nodule establishment identified after T-DNA transformation of *Lotus japonicus*. *Mol Gen Genet* 259**,** 414-423.

Stracke, S., Kistner, C., Yoshida, S., Mulder, L., Sato, S., Kaneko, T., Tabata, S., Sandal, N., Stougaard, J., Szczyglowski, K., and Parniske, M. (2002). A plant receptor-like kinase required for both bacterial and fungal symbiosis. *Nature* 417**,** 959-962. doi: 10.1038/nature00841.

Szczyglowski, K., Shaw, R.S., Wopereis, J., Copeland, S., Hamburger, D., Kasiborski, B., Dazzo, F.B., and De Bruijn, F.J. (1998). Nodule organogenesis and symbiotic mutants of the model legume *Lotus japonicus*. *Molecular Plant-Microbe Interactions* 11**,** 684-697. doi: 10.1094/MPMI.1998.11.7.684.
